# Metabolomics and proteomics analyses of Chrysanthemi Flos: a mechanism study of changes in proteins and metabolites by processing methods

**DOI:** 10.1186/s13020-024-01013-w

**Published:** 2024-11-19

**Authors:** Wei Zhang, Yu-wen Qin, Yang-fei Ding, Jun-wei Xiong, Xiang-wei Chang, Hong-su Zhao, Cheng-kai Xia, Jiu-ba Zhang, Yu Li, Chun-qin Mao, Tu-lin Lu, De-ling Wu

**Affiliations:** 1 College of Pharmacy, Nanjing University of Chinese Medicine, 138 Xianlin Rd, Nanjing, 210023 People’s Republic of China; 2 School of Pharmacy, Anhui University of Chinese Medicine, 350 Shaoquan Rd, Hefei, 230012 People’s Republic of China; 3 Anhui Province Key Laboratory of Traditional Chinese Medicine Decoction Pieces of New Manufacturing Technology, Hefei, 230012 China; 4 MOE-Anhui Joint Collaborative Innovation Center for Quality Improvement of Anhui Genuine Chinese Medicinal Materials, Hefei, 230012 China; 5 Joint Research Center for Chinese Herbal Medicine of Anhui of IHM, Bozhou Vocational and Technical College, Bozhou, 236800 China

**Keywords:** Chrysanthemi Flos, Traditional Chinese medicine, Proteomics, Metabolomics, Post-harvest processing, Drying methods

## Abstract

**Background:**

Chrysanthemi Flos is a traditional Chinese medicine with a long history of medicinal use. Prior research suggests that the intrinsic composition of Chrysanthemi Flos is affected by shade-drying and oven-drying methods. Nevertheless, the effects of these methods on the proteins and metabolites of Chrysanthemi Flos have not been extensively studied.

**Methods:**

The TMT (tandem mass tag) quantitative proteomics method and the LC–MS/MS (liquid chromatography-tandem mass spectrometry) non-targeted metabolomics method were used to systematically study the differences in the proteins and metabolites during the process of drying Chrysanthemi Flos in the shade and an oven.

**Results:**

Differentially accumulated metabolites and abundant proteins were primarily enriched in the purine metabolism, pyrimidine metabolism, cyanogenic amino acid metabolism, phenylpropanoid biosynthesis, and starch and sucrose metabolism pathways. Primary metabolites, such as guanine, xanthine, cytidine 5'-diphosphate serine, L-isoleucine, stearidonic acid, alginate, and inulin, play a crucial role in providing energy for Chrysanthemi Flos to withstand desiccation stress. The upregulation of ferulate-5- hydroxylase (F5H), shikimate O hydroxycinnamoyltransferase (HCT), caffeoyl-CoA O-methyltransferase (CCoAOMT), and chalcone isomerase (CHI) enzymes promotes the synthesis of flavonoids, including sinapic acid, caffeoyl shikimic acid, and naringenin chalcone, which possess antioxidant properties. Despite the notable improvements in energy metabolism and antioxidant capacity, these enhancements proved insufficient in halting the senescence and ultimate demise of Chrysanthemi Flos. Moreover, the shade-drying method can inhibit protein expression and promote the accumulation of bioactive components, but the drying efficiency is low, while the oven-drying method exhibits rapid drying efficiency, it does not effectively preserve the components.

**Conclusion:**

Our study offers a comprehensive explanation for the changes in protein expression and metabolite conversion observed in shade-dried and oven-dried Chrysanthemi Flos, also providing a foundation for optimizing the drying process of Chrysanthemi Flos.

**Supplementary Information:**

The online version contains supplementary material available at 10.1186/s13020-024-01013-w.

## Introduction

Chrysanthemi Flos is the dried flower head of a plant from the Asteraceae family that mainly consists of flavonoids, phenylpropanoids, and volatile oils. Recent research suggests that Chrysanthemi Flos has antimicrobial, anti-inflammatory, hypolipidemic, antitumor, and antioxidant properties. Chrysanthemi Flos has been used in Asian countries, especially China, for over 2000 years due to its wide range of flavor and medicinal properties [1]. According to the *Chinese Pharmacopoeia*, Chrysanthemi Flos is classified as *“Boju”*, *“Chuju”*, *“Gongju”*, *“Hangju”*, or *“Huaiju”* based on its origin and processing method [2].

Chrysanthemi Flos is a commonly used medicinal herb, often consumed as a floral tea with remarkable properties for soothing the throat and reducing inflammation. The herb's effectiveness in eliminating “fire” from the body makes it a favored option for natural remedies. However, freshly harvested Chrysanthemi Flos needs to be dried promptly, and the drying procedure plays a vital role in maintaining the quality and distinguishing it as an authentic medicinal Chrysanthemi Flos product. The common methods for drying Chrysanthemi Flos include oven drying, shade drying, pulsed vacuum drying, hot air drying, and microwave hot air drying [1,3]. Shade drying is the most commonly used method as it does not require expensive equipment, and the lower temperature helps prevent the degradation of the active components. On the other hand, oven drying is another commonly used technique for drying herbs on an industrial scale. While it reduces drying time and maintains uniformity, the use of high temperatures decreases the effectiveness of Chrysanthemi Flos [1].

The composition and content of proteins and chemical components in Chrysanthemi Flos are markedly influenced by different drying methods. When Chrysanthemi Flos is dried using steam kill-enzyme torrefaction, the levels of flavonoids and chlorogenic elements show a notable rise as the duration of steam and enzyme torrefaction increases. On the other hand, the amounts of free amino acids and soluble sugars initially increase but then decrease. In contrast, when kill-enzyme torrefaction is employed, the initial increase in flavonoid, chlorogenic acid, and vitamin C levels is followed by a decrease as the duration of the enzyme torrefaction increases, but the content of soluble sugars exhibits a significant increase [4]. However, when pulsed vacuum drying is employed, the Chrysanthemi Flos' overall phenolic and flavonoid content was reported to be preserved, and its antioxidant activity increased under a shorter duration [5]. Our initial investigation compared the impacts of various drying methods on the phenolic acids and flavonoids in Chrysanthemi Flos [6]. The research revealed that the quantity of flavonoids in *“Boju”* was mainly affected by the drying temperature. Shade drying resulted in higher levels of flavonoid glycosides and phenolic acids. We also determined that the β-glucosidase in Chrysanthemi Flos contributes to quality development, as per the "enzyme inhibition and glycoside preservation" processing principle. Limited research has been conducted to date on the alteration of metabolites and protein expression in Chrysanthemi Flos using two drying methods: shade drying and oven drying.

In this research, it is postulated that postharvest processing is an abiotic stress and that many of Chrysanthemi Flos’s own major sensors detect complex stimuli that lead to the production of various proteins and ultimately metabolic changes. For verification of this hypothesis, a comprehensive study of postharvest processing is necessary. Chrysanthemi Flos is in a low-temperature drying environment during shade drying and a high-temperature drying environment during oven drying, because metabolites are the end products of cellular regulatory processes, and the internal metabolites of Chrysanthemi Flos respond to its own cellular responses to environmental stresses under different drying conditions. Therefore, metabolomics can be used to study the overall metabolic changes before and after processing. In addition, protein expression can influence the amounts of active constituents, proteomics analyses of proteins involved in metabolite production and degradation were performed to better understand the molecular mechanisms that regulate the metabolism of active constituents. Therefore. From the perspective of the Chrysanthemi Flos itself in response to the drying environment, we conducted metabolomics and proteomics analyses in combination to explore the reasons for the changes in Chrysanthemi Flos under shade drying and oven drying. Our research will provide guidance for the systematic processing of Chrysanthemi Flos. To our knowledge, this is the inaugural application of a multi-omics approach to explore the molecular mechanisms underlying protein and metabolite alterations in Chrysanthemi Flos subjected to various drying methods.

## Materials and methods

### Chemicals and reagents

The chemicals and solvents, specifically methanol, formic acid, water, and acetonitrile, were obtained from Thermo Fisher Scientific Co., Ltd. (Thermo Fisher, USA), while L-2-chlorophenylalanine was obtained from Shanghai Hengchuang Biotechnology Co., Ltd. (Shanghai, China). Isochlorogenic acid A (batch no. DSTDY003603), isochlorogenic acid B (batch no. DSTDY003703), isochlorogenic acid C (batch no. DSTDY003804), neochlorogenic acid (batch no. DSTDX001504), cryptochlorogenic acid (batch no. DST220104-035), and chlorogenic acid (batch no. DSTDL002103) were obtained from Chengdu DeSiTe Biological Technology Co., Ltd. (China). Nicotiflorin (batch no. CFS202301) was purchased from Wuhan Economic & Technological Development Zone (China). Emodin was obtained from Shanghai yuanye Bio-Technology Co., Ltd. (China). Ultrapure water was obtained from a Milli-Q water purification system (Millipore, USA). All the previously mentioned substances were of analytical purity or chromatographic grade.

### Plant materials

A total of 8 batches of Chrysanthemi Flos samples were procured from Qiaocheng District, Bozhou city, Anhui Province, and authenticated as *Chrysanthemum morifolium* (Ramat.) Tzvel. *“Boju”* by Professor Nianjun Yu from the School of Pharmacy at Anhui University of Chinese Medicine. The samples were assigned the identification number BJ20211116 and are currently stored at the Department of Traditional Chinese Medicine and Natural Medicine, Anhui University of Chinese Medicine. Upon picking the Chrysanthemi Flos, fresh Chrysanthemi Flos (FCF) samples were obtained and immediately frozen at -80 °C until analysis. Alternatively, the freshly harvested Chrysanthemi Flos were dried at 50 °C in an oven (DCF) for 20 h to produce the DCF samples. An alternative approach involved drying the harvested Chrysanthemi Flos in the shade (SCF) for 2 to 3 weeks in a flat, ventilated area shielded from light to obtain the SCF samples. Prior to analysis, the samples were subjected to storage at a temperature of -80℃ and subsequently pulverized into a fine powder using liquid nitrogen as a biological replicant. Both metabolomics and proteomics analysis were conducted using eight biological replicates.

### Proteomics analysis

#### Protein extraction, digestion, and TMT labeling

The frozen samples were rapidly ground into a fine, consistent powder using liquid nitrogen. Subsequently, the powder was homogenized in 1 mL of phenol extraction buffer (Solarbio Life Sciences, Beijing, China), followed by the addition of 1 mL of saturated phenol and Tris HCl (pH 7.8) (Sangon Biotech (Shanghai) Co, Ltd., Shanghai, China). The mixture was agitated and held at 4 °C for 30 min. The upper phenol phase was separated from the aqueous phase by centrifugation at 4 °C for 10 min at 7100*g*. The isolated upper phenol phase was then combined with five times the volume of pre-cooled 0.1 M ammonium acetate methanol (Shanghai Anpel Experiment Technologies Co, Ltd., Shanghai, China). The precipitated protein was pelleted by centrifugation at 12,000*g* and 4 °C for 10 min, followed by overnight incubation at ± 20 °C. The purification process involved two rounds of particle resuspension in pre-cooled methanol and ice-cooled acetone (BJOKA Biotech Co, Ltd., Beijing, China), followed by another round of centrifugation. The collected particles were air-dried, suspended in 300 μL of pyrolysis solution, and incubated at room temperature for 3 h. The resulting solution was centrifuged to remove any insoluble fractions, resulting in a supernatant containing total extractable protein.

Based on the quantified protein concentration, equivalent quantities of protein were extracted from each sample, and distinct sample groups were subsequently diluted to identical concentrations and volumes. The protein solution was treated with 25 mM dithiothreitol (DTT) from Shanghai Titan Scientific Co., Ltd. (Shanghai, China), and incubated at 55 °C for 30 to 60 min to reduce the DTT concentration to approximately 5 mM. Following this, a stoichiometric amount of iodoacetamide, supplied by Sangon Biotech (Shanghai) Co., Ltd. (Shanghai, China), was introduced to reach an approximate concentration of 10 mM. The solution was then incubated in the dark at room temperature for 15 to 30 min. To precipitate the proteins, six volumes of precooled acetone were added, and the mixture was subsequently stored at – 20 °C for a minimum of four hours or overnight. The precipitate was collected by centrifugation at 8000*g* and 4 °C for 10 min. For enzymatic digestion, an enzymatic diluent was added to the protein at a ratio of 50:1 (w/w), equating to 100 μg of protein to 2 μg of enzyme. The mixture was then solubilized and incubated at 37 °C for 12 h. Post-digestion, the sample was subjected to lyophilization.

For TMT labeling, the lyophilized samples were dissolved in 50 μL of 100 mM triethylammonium bicarbonate (TEAB) provided by Sangon Biotech (Shanghai) Co., Ltd. (Shanghai, China). The labeling was conducted in 1.5 mL Eppendorf tubes. To prepare the TMT reagent, 20 μL of acetonitrile was added to its container at room temperature, followed by vortexing and a brief centrifugation at 5 min to ensure complete dissolution. This step was repeated to guarantee thorough mixing. Then, 10 μL of the TMT reagent was added to each sample, which was subsequently mixed and incubated at room temperature for 1 h. To terminate the labeling reaction, 5 μL of 5% hydroxylamine was introduced to each sample and incubated for an additional 15 min. The labeled peptide solutions were then lyophilized and stored at -80 °C until further analysis.

#### LC–MS/MS analysis

An Orbitrap Fusion Mass Spectrometer (Thermo Fisher, USA) equipped with a nano-spray Flex source (Thermo Fisher, USA) was utilized for all analyses. The samples were uploaded onto an Acclaim PepMap 100 C_18_ LC column (3 μm, 100 μm × 2 cm, Thermo Fisher, USA) at a flow rate of 300 nL/min on the Easy-nLC 1200 system platform (Thermo Fisher, USA) and separated on an analytical Acclaim PepMap RSLC C_18_ column (2 μm, 75 μm × 50 cm, Thermo Fisher, USA) with a linear gradient (0–1 min, 5–8% B; 1–31 min, 8–30% B; 31–46 min, 30–50% B; 46–61 min, 50–100% B; 61–68 min, 100% B). The mobile phase consisted of A = 0.1% FA in water and B = 0.1% FA and 19.9% H_2_O in ACN. The mass spectrometer was operated in data acquisition mode with Xcalibur v.4.0 (Thermo Fisher, USA). A comprehensive mass spectrometry (MS) scan was conducted within the mass range of 375–1500 *m/z*, with a mass resolution of 60,000 and an automatic gain control (AGC) target value of 4e^5^. The 20 most intense peaks in the MS spectrum were subjected to higher energy collision dissociation (HCD) fragmentation, utilizing a collision energy of 38. The MS/MS spectrum exhibited a resolution of 50,000, an AGC target of 1e^5^, and a maximum injection time of 120 ms. The Orbitrap Fusion Dynamic Exclusion was configured to operate in forward mode with a duration of 60.0 s.

#### Database search and data analysis

The MS/MS peptide data were studied using Proteome Discoverer v.2.4 (Thermo Fisher, USA), which was then matched to the Uniprot-Taxonomy_886714.Asta database. TMT was chosen as the protein quantification method. The search parameters were specified as follows: a precursor ion mass tolerance of 10 ppm, a fragment ion mass tolerance of 0.02 Da, static modifications including TMT labeling at the N-terminus and lysine (K) residues, and carbamidomethylation of cysteine (C) residues. Dynamic modifications encompassed methionine oxidation and N-terminal acetylation. Trypsin digestion was allowed up to two missed cleavages. Protein identifications required the presence of at least one uniquely identified peptide. The false discovery rate (FDR) for peptide hits was set at 0.01 or lower. Proteins exhibiting a differential abundance with a fold change of two or more (≥ 2.0 or ≤ 0.5) and a P-value of 0.05 or less were considered significant. Functional classification and pathway analysis of the differentially abundant proteins were conducted using the Gene Ontology (GO) database, the clusters of orthologous groups for eukaryotic complete genomes (KOG), and the Kyoto Encyclopedia of Genes and Genomes (KEGG) database.

### Non-targeted metabolomics analysis

#### Metabolite extraction

A quantity of 0.5 g of Chrysanthemi Flos pollen was precisely measured and subsequently dissolved in a 70% methanol/water solution (v/v) of 20 mL. The solution underwent ultrasonic treatment on ice at a power of 400 W and a frequency of 40 kHz for a duration of 1 hour. The resulting mixture was subsequently centrifuged at 13,000 × *g* for 15 minutes at 4°C. The LC–MS analysis of the supernatant was conducted, and quality control (QC) samples were prepared by combining equivalent quantities of supernatant derived from each Chrysanthemi Flos sample extract. To guarantee the consistency of the analysis, a total of 6 QC samples were added to the test sequence, which consisted of 24 samples. In short, a QC sample was added at intervals of 4 samples. Moreover, we have also added mixed standard solutions of suitable concentration to QC-2 in the QC sample cohort to ensure the accuracy of metabolite identification.

#### UHPLC-MS/MS analysis and preliminary identification of metabolites

The UHPLC-MS/MS analysis was conducted using a combination of UHPLC (AB ExionLC, AB Sciex, USA) and QE plus (Thermo Fisher, USA). The samples were subjected to analysis on an Accucore C_18_ column (2.6 μm, 2.1 × 100 mm, Thermo Fisher, USA). The binary gradient elution system comprised acetonitrile (with 0.1% formic acid, v/v) and water (with 0.1% formic acid, v/v). The separation was accomplished by implementing the following gradient conditions: 0 min, 95% B; 2 min, 90% B; 6 min, 80% B; 11 min, 79% B; 16 min, 74% B; 20 min, 70% B; 23 min, 50% B; 26 min, 40% B; 28 min, 30% B; 30 min, 5% B; 31 min, 95% B; and 33 min, 95% B. The experimental conditions included a flow rate of 0.20 mL/min, a column temperature maintained at 30 °C, and sample storage at 4 °C during the analysis period. An injection volume of 2 μL was utilized, and MS data acquisition was performed in both positive and negative ion modes using electrospray ionization (ESI). The MS parameters were set to a scan range of 100–1500 *m/z*, a spray voltage of 3800 V for positive and 3000 V for negative modes, a capillary temperature of 320 °C, an auxiliary gas heater temperature of 350 °C, a sheath gas flow rate of 35 arbitrary units (arb), an auxiliary gas flow rate of 5 arb, and an S-lens RF level of 50. Fragment collision energies were normalized to 10, 20, and 40 electron volts. Resolutions were set at 70,000 for full MS scans and 17,500 for MS^2^ scans. Data processing involved baseline filtering, peak identification, integration, retention time correction, peak alignment, and normalization, conducted using Progenesis QI v.2.3 (Nonlinear Dynamics, Newcastle, UK), with set parameters of a 5 ppm precursor tolerance, a 10 ppm product tolerance, and a product ion threshold of 5%. Metabolites identification was based on precise mass-to-charge ratios (*m/z*), secondary fragmentation and isotopic distribution. Component databases are used for qualitative analyses, including the Human Metabolome Database (HMDB, https://www.hmdb.cal), Lipidmaps (http://www.lipidmaps.org/), Metlin (https://metlin.scripps.edu/) and self-built databases (OE Biotech Co., Lid., Shanghai, China).

#### Multivariate statistical analysis and pathway analysis

The first step in the multivariate statistical analysis is the application of unsupervised principal component analysis (PCA) to assess the overall distribution of samples and to verify the consistency of the analytical process. Subsequently, supervised orthogonal partial least squares discriminant analysis (OPLS-DA) is utilized to discern the variations in metabolic profiles between groups and to pinpoint metabolites with differential expression among these groups.

The OPLS-DA model yielded a statistically significant variable influence threshold of projection value (VIP), and the metabolites exhibiting significant differences were identified through the application of Student's t-test (P-value) to the original data. Specifically, metabolites with a VIP score of 1.0 or higher and a P-value of 0.05 or lower were considered statistically significant. Additionally, metabolites displaying significant abundance variations underwent further screening based on their fold change (FC), with thresholds set at 2.0 or higher, or 0.5 or lower. The differential accumulation of metabolites was further examined through the KEGG database to elucidate metabolic pathways.

## Results

### Metabolic profiles of Chrysanthemi Flos samples processed using different methods

#### Qualitative analysis of metabolites

Non-targeted LC–MS/MS analysis was performed employing ESI + and ESI− modes. Fig. S1 displays a representative total ion chromatogram (TIC) of Chrysanthemi Flos samples subjected to various drying methods. The results revealed that the positive ion mode identified a larger array of compounds compared to the negative ion mode. Table S1 lists the metabolites’ names, their peak integration values, sequence numbers, and additional relevant data. In total, 3237 metabolites were preliminarily identified, with 3216 of these metabolites being annotated utilizing the HMDB. In addition, to verify the identification accuracy of metabolite identity, we focused on comparing the retention time (RT) and mass spectrometry information of cryptochlorogenic acid and chlorogenic acid identified in the negativeion mode of the mixed standard and Table S1. As illustrated in Fig. S2, the RT and secondary mass spectrometry information of cryptochlorogenic acid and chlorogenic acid in the mixed standard and the samples remained consistent, indicating the reliability of our metabolite identification results for subsequent analysis.

#### Multivariate statistical analysis of metabolites

**Fig. 1:**
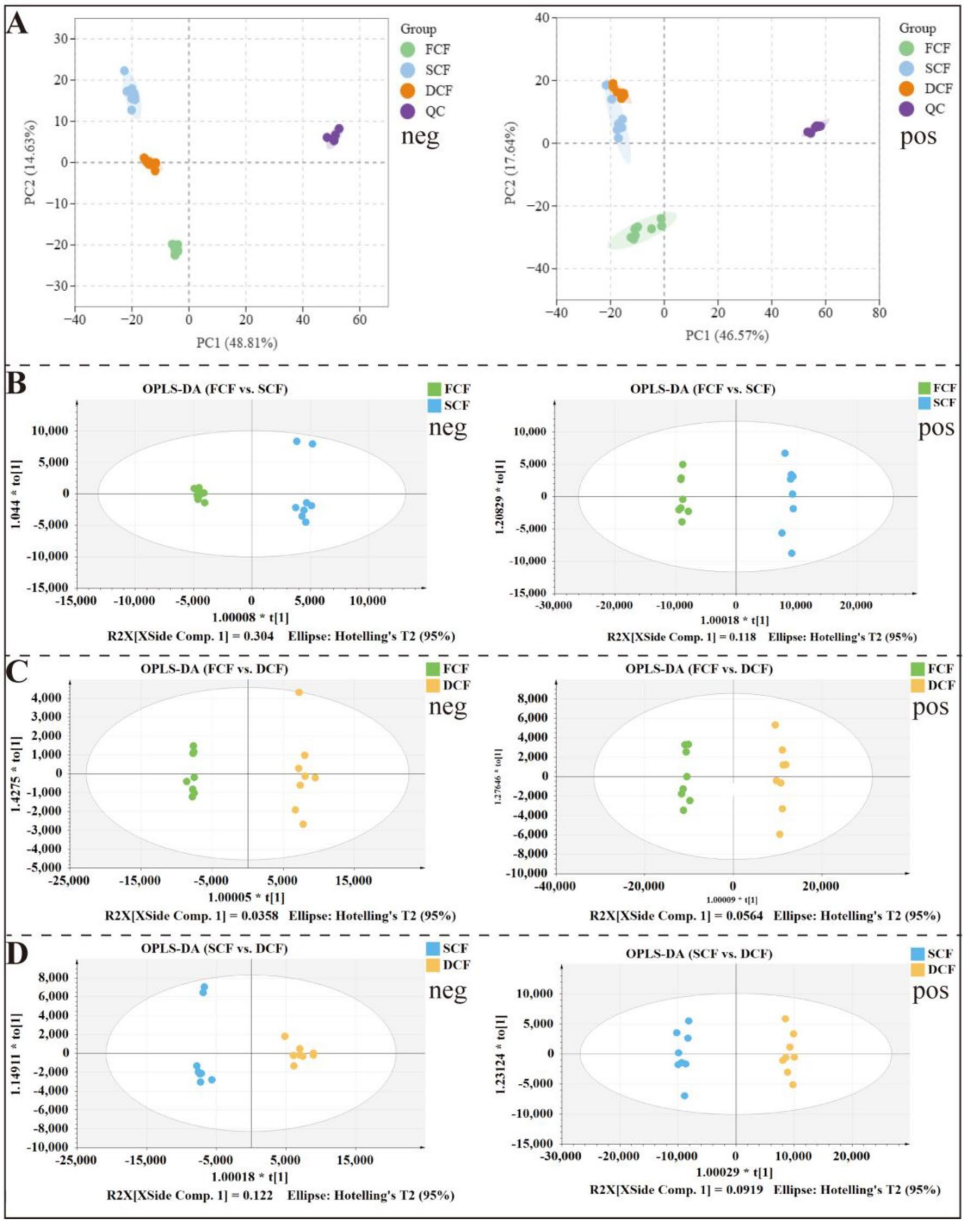
Multivariate statistical analysis of Chrysanthemi Flos samples. **A** PCA results (metabolomics); **B** OPLS-DA results (FCF vs. SCF group); **C** OPLS-DA results (FCF vs. DCF group); **D** OPLS-DA results (SCF vs. DCF group)

Multivariate statistical analyses, including PCA and OPLS-DA, were utilized to simplify the complexity of the metabolite data and improve the interpretability and robustness of the findings. PCA was performed on the samples to detect differences in metabolites among and within groups. The results showed the clear separation of the FCF, SCF, and DCF samples into three distinct groups on the PCA charts (Fig. 1A, B). The QC samples overlapped, indicating the reliability and consistency of the metabolomic analyses. In addition, we performed supervised classification using the OPLS-DA model to evaluate the degree of metabolite transformation across different Chrysanthemi Flos samples. The OPLS-DA plots (Fig. 1B–D) revealed substantial variation between the sample groups, indicating pronounced differences among them. Furthermore, our experiments showed favorable model parameters. The OPLS-DA model did not indicate overfitting after the 200-permutation test (Fig. S3). Therefore, the metabolomics data were suitable for further analysis.

#### Screening and classification of differentially accumulated metabolites

**Fig. 2:**
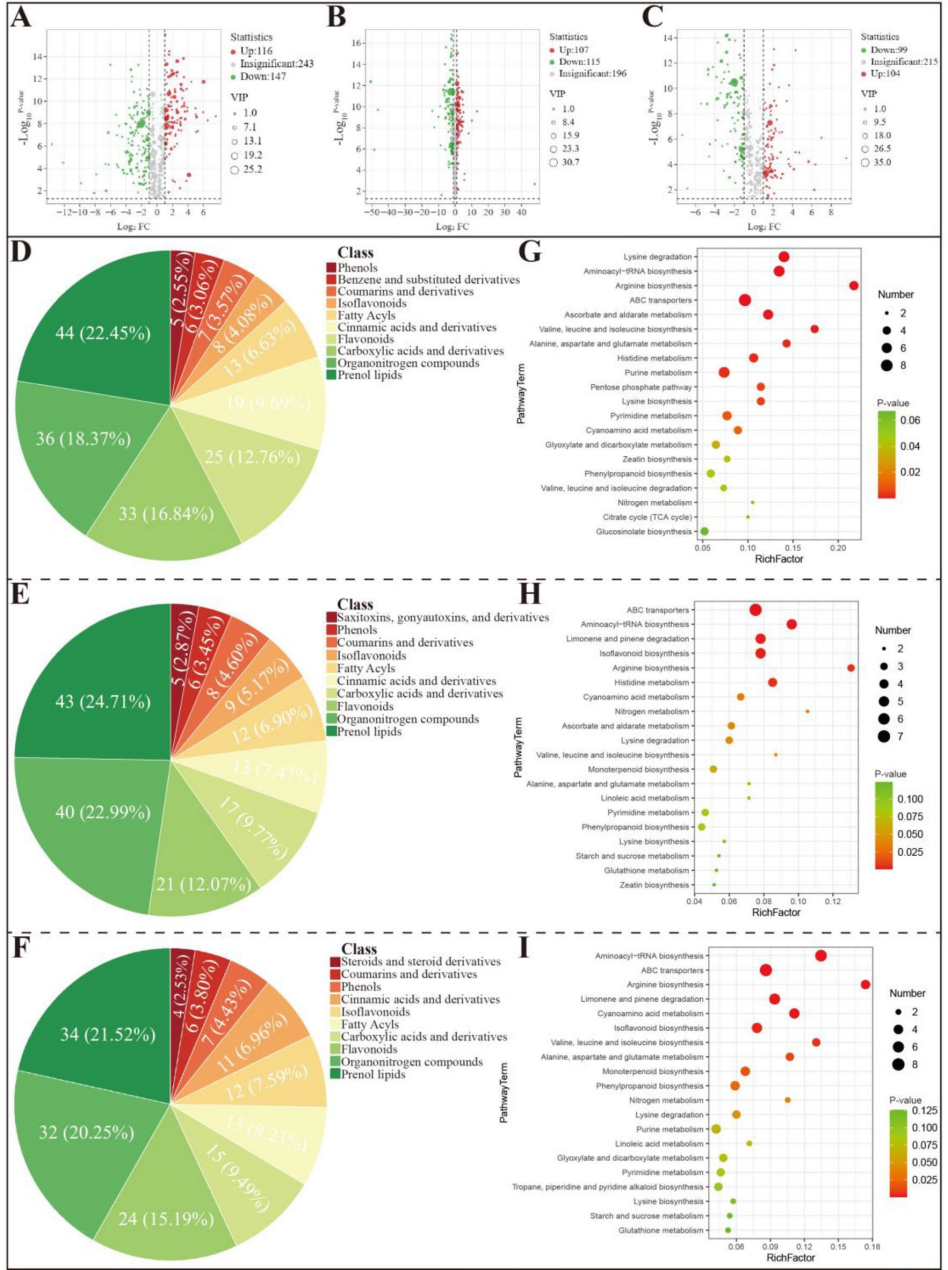
**A**–**C** Volcano plots of differentially accumulated metabolites: FCF vs. SCF group, FCF vs. DCF group, and SCF vs. DCF group, respectively. **D**–**F** TOP10 classification charts of differentially accumulated metabolites: FCF vs. SCF group, FCF vs. DCF group, and SCF vs. DCF group, respectively. **G**–**I** Metabolomics pathway enrichment analysis results: FCF vs. SCF group, FCF vs. DCF group, and SCF vs. DCF group, respectively

Table S2 presents a comparison of the metabolites from FCF, SCF, and DCF. Metabolomic analysis indicated differential accumulation of 506 metabolites between FCF and SCF, with 116 up-regulated and 147 down-regulated, as shown in Fig. 2A. Similarly, 418 metabolites differed between FCF and DCF, with 107 up-regulated and 115 down-regulated (Fig. 2B). Between SCF and DCF, 418 metabolites exhibited differential accumulation, with 99 up-regulated and 104 down-regulated (Fig. 2C). These metabolites were cross-referenced with the HMDB for annotation. As shown in Fig. 2D–F, the primary categories of differentially accumulated metabolites from dried Chrysanthemi Flos were prenol lipids, organooxygen compounds, and flavonoids.

#### Metabolite accumulation patterns and pathway analysis

Enrichment analyses of metabolic pathways offer insights into the underlying mechanisms driving metabolic alterations across varied samples. The KEGG database was used to assess the enrichment of metabolic pathways for different metabolites, and the pathway enrichment results are shown in Table S3. The top 20 distinct metabolic pathways were visually analyzed to compare FCF, SCF, and DCF (Fig. 2G–I). In the FCF and SCF comparison, 17 KEGG pathway terms displayed significantly different enrichment profiles (P-value < 0.05), including lysine degradation, aminoacyl-tRNA biosynthesis, arginine biosynthesis, ABC transporters, ascorbate and aldarate metabolism, valine, leucine, and isoleucine biosynthesis, alanine, aspartate, and glutamate metabolism, histidine metabolism, purine metabolism, lysine biosynthesis, pentose phosphate pathway, pyrimidine metabolism, cyanoamino acid metabolism, glyoxylate and dicarboxylate metabolism, zeatin biosynthesis, phenylpropanoid biosynthesis, and valine, leucine, and isoleucine degradation. A total of 11 KEGG pathway terms showed significantly different enrichment profiles when FCF and DCF were compared (P-value < 0.05). These terms included ABC transporters, aminoacyl-tRNA biosynthesis, isoflavonoid biosynthesis, limonene and pinene catabolism, arginine biosynthesis, histidine metabolism, cyanoamino acid metabolism, nitrogen metabolism, ascorbate and aldarate metabolism, lysine catabolism, and valine, leucine, and isoleucine biosynthesis. In the SCF and DCF comparison, 12 KEGG pathway terms demonstrated significantly different enrichment patterns (P-value < 0.05). These pathways included aminoacyl-tRNA biosynthesis, ABC transporters, arginine biosynthesis, limonene and pinene degradation, cyanoamino acid metabolism, isoflavonoid biosynthesis, valine, leucine, and isoleucine biosynthesis, alanine, aspartate, and glutamate metabolism, monoterpenoid biosynthesis, phenylpropanoid biosynthesis, nitrogen metabolism, and lysine degradation.

### Proteomic analysis of differently processed Chrysanthemi Flos samples

#### Identification and analysis of proteins

**Fig. 3:**
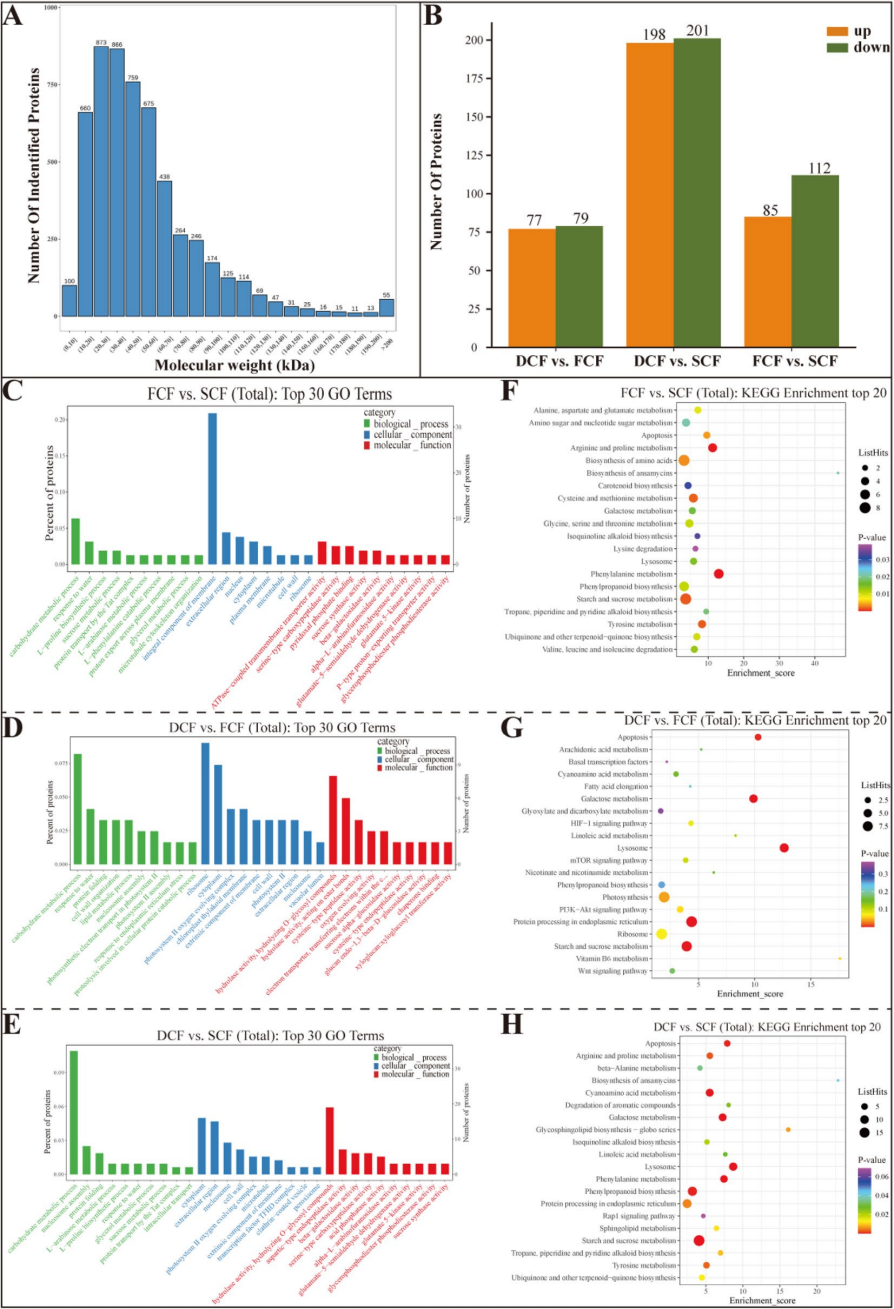
**A** Molecular mass distribution diagram. **B** Differential protein statistical diagram. **C** GO enrichment analysis (FCF vs. SCF). **D** GO enrichment analysis (DCF vs. SCF). **E** GO enrichment analysis (DCF vs. FCF). **F** KEGG enrichment analysis (FCF vs. SCF). **G** KEGG enrichment analysis (DCF vs. FCF). **H** KEGG enrichment analysis (DCF vs. SCF)

In our study, 333,220 MS/MS spectra were identified, of which 33,018 corresponded to identified MS spectra (Fig. S4). A total of 21,099 peptides were identified, with 17,645 being distinct. Ultimately, the use of at least one non-recurring peptide led to the discovery of no fewer than 6010 proteins across all samples. The detected polyproteins mainly had a relative molecular weight ranging from 10 to 60 kDa, as depicted in Fig. 3A.

To analyze the differences among Chrysanthemi Flos samples processed with different drying methods, the complete proteomics data underwent principal component analysis. The proteomics PCA results (Fig. S5) demonstrate a clear distinction among the Chrysanthemi Flos samples. The quantities of up- and down-regulated proteins between FCF, SCF, and DCF are presented in Fig. 3B. The proteins that exhibited differential abundance in each sample are listed in Table S4. In total, 156 proteins, with 77 upregulated and 79 downregulated, were detected in both FCF and DCF. Moreover, 399 proteins were identified in DCF and SCF, with 198 being up-regulated and 201 being down-regulated. Similarly, FCF and SCF revealed the identification of 197 proteins, with 85 being up-regulated and 112 being down-regulated. Compared to FCF, the decrease in specifically enriched proteins in DCF was more pronounced than the decrease in up-regulated proteins. This suggests that the drying methods resulted in complex metabolic pathway regulation in Chrysanthemi Flos.

#### Categorization and annotation of proteins with differential abundance

The differential protein GO enrichment analysis results for the different dried Chrysanthemi Flos samples are shown in Fig. 3C–E. The differentially expressed proteins were classified according to their functional attributes into three categories: biological processes (BP), cellular components (CC), and molecular functions (MF).

The BP category revealed that the primary functional categories of various dried Chrysanthemi Flos types encompassed “carbohydrate metabolism processes”, “response to water”, “L-proline biosynthesis processes”, “sucrose metabolism processes”, and “protein transport by Tat complex” for both FCF and SCF. Additionally, DCF and SCF exhibited “carbohydrate metabolism process”, “nucleosome assembly”, “protein folding”, “L-arabinose metabolism process”, and “L-proline biosynthesis process”. Finally, DCF and FCF demonstrated “carbohydrate metabolic process”, “response to water, protein folding”, “cell wall organization”, and “lipid metabolic process”.

The CC class encompassed a variety of functional classes, including “integral components of membrane”, “extracellular region”, “nucleus”, “cytoplasm”, and “plasma membrane”, which were present in both FCF and SCF. Additionally, the “cytoplasm”, “extracellular region”, “nucleosome”, “cell wall”, and “photosystem II oxygen evolving complex” were observed in DCF and SCF, while the “ribosome”, “cytoplasm”, “photosystem II oxygen evolving complex”, “chloroplast thylakoid membrane”, and “extrinsic component of membrane” were present in DCF and FCF.

The MF category encompasses several main functional categories, including "ATPase-coupled transmembrane transporter activity", "serine-type carboxypeptidase activity", "pyridoxal phosphate binding", "sucrose synthase activity", "beta-galactosidase activity" (found in both FCF and SCF); "hydrolase activity, hydrolyzing O-glycosyl compounds", "aspartic-type endopeptidase activity", "beta-galactosidase activity", "serine-type carboxypeptidase activity", "acid phosphatase activity", and "α-L-arabinofuranosidase activity" (found in both DCF and SCF); and "hydrolase activity, hydrolyzing O-glycosyl compounds", "hydrolase activity, acting on ester bonds", "cysteine-type peptidase activity", "oxygen evolving activity", and "electron transporter, transferring electrons within the cyclic electron transport pathway of photosynthesis activity" (found in both DCF and FCF).

#### Pathway enrichment analysis for proteins with differential abundance

The KEGG database was employed to visually represent the various protein profiles. As illustrated in Fig. 3F–H. The KEGG signal pathway analysis revealed that the “biosynthesis of amino acids”, “starch and sucrose metabolism”, “phenylalanine biosynthesis”, “phenylpropanoid biosynthesis”, and “arginine and proline metabolism” were involved in different proteins in the FCF vs. SCF group. Additionally, the DCF vs. SCF group exhibited highly enriched pathways in terms of “starch and sucrose metabolism”, “phenylpropanoid biosynthesis”, “lysosome” and “phenylalanine metabolism”, which involve different proteins. Furthermore, the DCF vs. FCF group comparison displayed highly enriched pathways in terms of “photosynthesis”, “protein processing in the endoplasmic reticulum”, “starch and sucrose metabolism”, “lysosome”, and “galactose metabolism”. The pathways in question were significant in the metabolic changes of Chrysanthemi Flos during drying (Table S5).

### Convergence of metabolomic and proteomic analysis

**Fig. 4:**
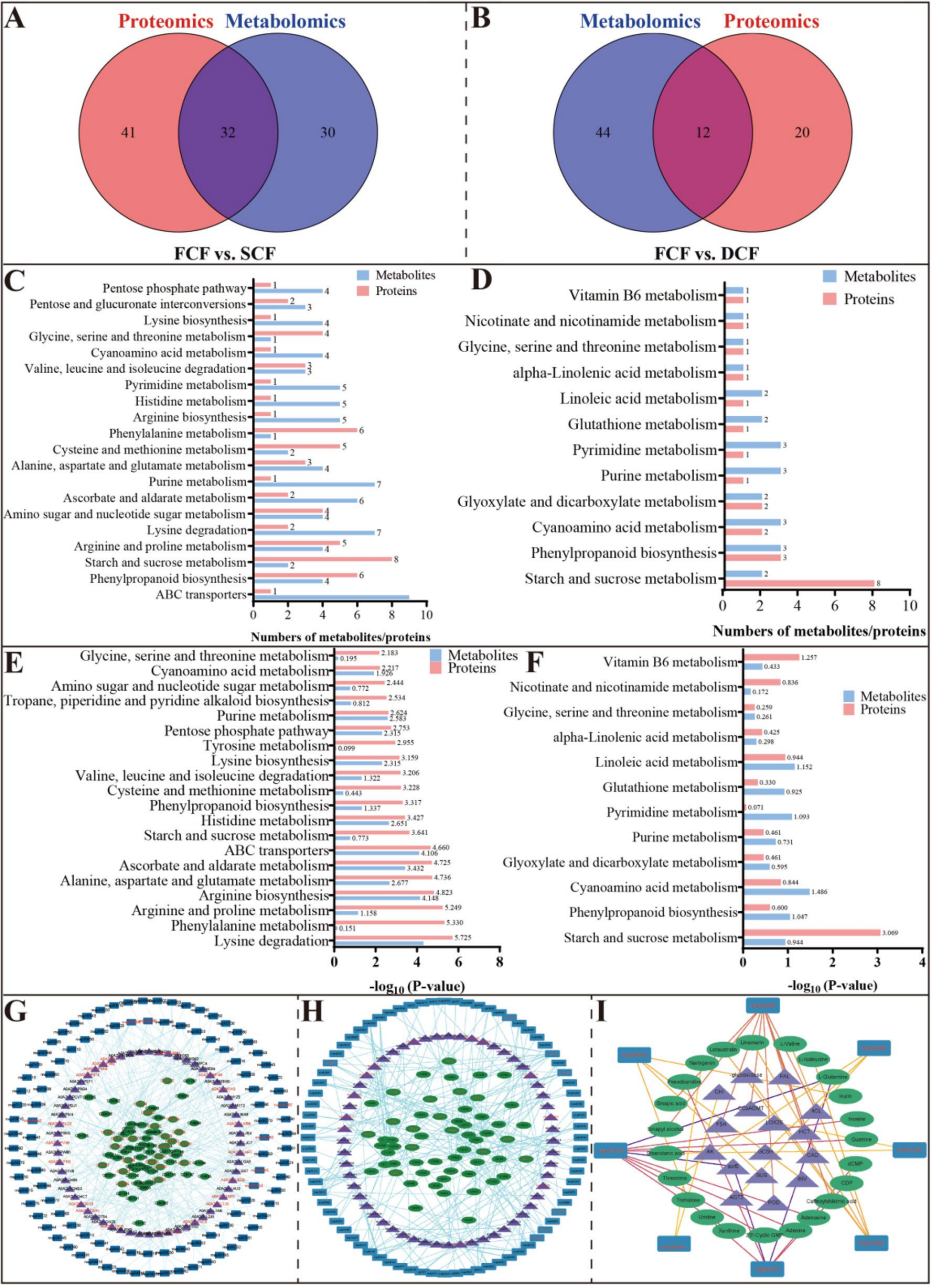
Combined proteomics–metabolomics analysis. **A**–**B** Venn diagrams of KEGG pathways involving different accumulated metabolites and enriched proteins. FCF vs. SCF and FCF vs. DCF, respectively. **C**–**D** The top 20 KEGG pathways containing the most metabolites and proteins. FCF vs. SCF and FCF vs. DCF, respectively. **E**–**F** The top 20 KEGG pathways enriched by metabolites and proteins (ranked by P-value). FCF vs. SCF and FCF vs. DCF, respectively. **G**–**H** Network analysis constructed for all differentially accumulated metabolites and enriched proteins. FCF vs. SCF and FCF vs. DCF, respectively. **I** Network analysis of metabolites and proteins involved in key pathways

To explore the effects of processing methods on the proteins and metabolites of Chrysanthemi Flos, the KEGG pathway was considered the carrier based on proteomics and metabolomics data and was selected for comprehensive analysis. Figure 4A, B displays the number of KEGG pathways associated with the proteome and metabolome. The overlapping sections of the Venn diagrams indicate the shared KEGG pathways between the differentially accumulated metabolites and the enriched proteins identified by both omics analyses. A comprehensive examination of metabolomic and proteomic data (refer to Table S5) revealed that FCF and SCF have 32 pathways in common, including flavonoid biosynthesis, phenylpropanoid biosynthesis, valine biosynthesis, leucine biosynthesis, and isoleucine biosynthesis. FCF and DCF shared 12 common metabolic pathways, including cyanoamino acid metabolism, linoleic acid metabolism, pyrimidine metabolism, and phenylpropanoid biosynthesis. Further analysis of the pathways shared between proteomics and metabolomics revealed the top 20 KEGG pathways with the greatest number of associated proteins and metabolites (Fig. 4C, D). These pathways are ranked by P-value in Fig. 4E, F, with a higher cumulative value from top to bottom suggesting increased biological pathway activity in the samples. Integration of proteomic and metabolomic data suggests that the identified metabolites and proteins are predominantly involved in primary and secondary metabolism.

Moreover, to elucidate the relationship between metabolomics and proteomics, network analysis was conducted on differentially accumulated metabolites, enriched proteins, and KEGG pathways using Cytoscape v.3.9.1 for FCF, SCF, and DCF. Figure 4G, H shows that seven biosynthetic pathways were affected by the shade-drying and oven-drying methods of processing Chrysanthemi Flos, i.e., cyanoamino acid metabolism, pyrimidine metabolism, starch and sucrose metabolism, phenylpropanoid biosynthesis, purine metabolism, glycine, serine and threonine metabolism, and α-Linolenic acid metabolism. A single metabolite may interact with multiple proteins, and conversely, one protein may bind to several metabolites within a shared pathway; thus, we integrated these pivotal metabolites and proteins with their corresponding pathways into a cohesive network. Figure 4I shows that the key pathway involved purine metabolism, pyrimidine metabolism, glycine, serine and threonine metabolism, cyanoamino acid metabolism, α-Linolenic acid metabolism, starch and sucrose metabolism, phenylpropanoid biosynthesis, and flavonoid biosynthesis. These findings suggest that various processing methods markedly influence purine metabolism, pyrimidine metabolism, glycine, serine, and threonine metabolism, cyanoamino acid metabolism, α-Linolenic acid metabolism, starch and sucrose metabolism, phenylpropanoid biosynthesis, and flavonoid biosynthesis in Chrysanthemi Flos.

## Discussion

As an important medicinal and dual-use flower tea, Chrysanthemi Flos is widely popular. It usually needs to be dried before being used. The drying processes include shade drying, in which fresh Chrysanthemi Flos heads are placed in a cool, ventilated location until completely dry, and there is oven drying, in which fresh Chrysanthemi Flos heads are placed in a 50 °C oven, followed by complete drying. Previous studies have confirmed that the choice of different drying methods for fresh Chrysanthemi Flos has significant effects on metabolites and enzymes [6]. However, the effects of drying Chrysanthemi Flos using shade drying or oven drying methods have not yet been elucidated.

**Fig. 5:**
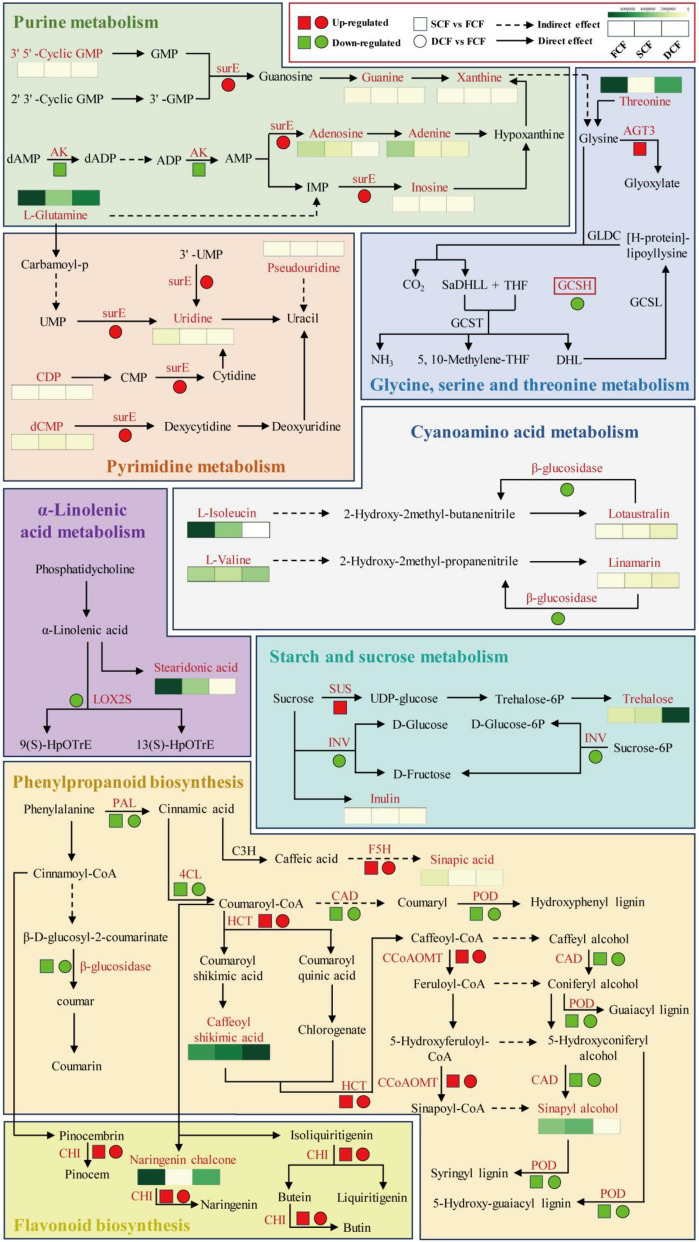
Changes in the biosynthesis of purine metabolism, pyrimidine metabolism, glycine, serine, and threonine metabolism, cyanoamino acid metabolism, α-Linolenic acid metabolism, starch and sucrose metabolism, phenylpropanoid biosynthesis, and flavonoid biosynthesis in the FCF, SCF, and DCF groups. Red symbolizes up-regulation, while green symbolizes down-regulation; squares represent the differential protein expression trend of SCF vs. FCF; circles represent the differential protein expression trend of DCF vs. FCF (SurE: 5′/3′-nucleotidase; AK: adenylate kinase; AGT3: alanine: glyoxylate aminotransferase 3; GCSH: glycine cleavage system H protein; CDP: cytidine 5'-diphosphate; dCMP: deoxycytidylic acid; LOX2S: lipoxygenase; SUS: sucrose synthase; INV: β-fructofuranosidase; PAL: phenylalanine ammonia-lyase; 4CL: 4-coumarate-CoA ligase; F5H: ferulate-5-hydroxylase; CAD: cinnamyl-alcohol dehydrogenase; HCT: shikimate O-hydroxycinnamoyltransferase; CCoAOMT: caffeoyl-CoA O-methyltransferase; POD: peroxidase; CHI: chalcone isomerase)

By comparing metabolomics and proteomics analyses, we found significant effects on primary and secondary metabolism when Chrysanthemi flos was processed via shade drying versus oven drying, which indicates that Chrysanthemi Flos undergoes complex metabolic regulation during the drying process. We described the differential regulation of key pathways (Fig. 5), including α-Linolenic acid metabolism, cyanogenic amino acid metabolism, glycine, serine, and threonine metabolism, phenylpropanoid biosynthesis, purine metabolism, pyrimidine metabolism, and starch and sucrose metabolism. The subsequent sections will discuss the functional classification of the accumulated metabolites and abundant proteins, as well as their associated metabolic reactions. These findings contribute to clarifying the intricate mechanisms of metabolic regulation in Chrysanthemi Flos throughout the drying process.

### Effects of drying methods on nucleotide metabolism in Chrysanthemi Flos

Nucleotides are one of the most important nitrogen compounds in any organism. The biosynthesis and metabolism of nucleotides are essential for plant growth and development. Purine and pyrimidine nucleotides are involved in many biochemical processes in plants. They are metabolites involved in bioenergetic processes and the synthesis of macromolecules including polysaccharides, phospholipids, and glycolipids [7]. In our study, we found that Chrysanthemi Flos showed a down-regulation of nucleotide metabolites, including 3′ 5′-Cyclic GMP, Guanine, Xanthine, L-Glutamine, Adenosine, Adenine, Inosine, Uridine, CDP, and dCMP. In the SCF and FCF comparison, AK expression was found to be down-regulated. In contrast, SurE was significantly up-regulated in DCF vs. FCF.

AK, an enzyme widespread across diverse life forms, facilitates the reversible transphosphorylation (2ADP ↔ ATP + AMP), essential for energy metabolism regulation and adenylate pool equilibrium [8]. Additionally, AK is instrumental in plant responses to environmental stressors [9, 10]. From our results, we found that AK was markedly down-regulated in SCF vs. FCF, which may be due to the reduction of its own energy consumption to maintain normal physiological activities in response to the stressful effects of low-temperature and low-drying environments during shade drying. However, prolonged darkness enhances purine catabolism, which gradually supplies the normal activities of mitochondria, chloroplasts, and other tissue cells by enhancing nucleotide metabolism [11]. During the process of drying in the shade, Chrysanthemi Flos is actually in a slow physiological process of "dying", gradually consuming nucleotide compounds to supply its own energy consumption to resist this abiotic stress response, which is in accordance with our metabolomics data.

We found that the SurE of Chrysanthemi Flos was up-regulated during the oven-drying process. SurE efficiently catalyzes the dephosphorylation of 5'-ribonucleotides and 5'-deoxyribonucleotides, yielding nucleosides and inorganic phosphate, and demonstrates broad specificity by acting on various phosphorylated substrates. SurE can be involved in physiological processes such as purine and pyrimidine salvage pathways, inter-cellular signaling, and the control of ribonucleotide and deoxyribonucleotide pools [12]. The up-regulation of SurE in DCF vs. FCF may have resulted from the fact that DCF was exposed to high temperatures, which could resist this high-temperature drought stress response by promoting the metabolism of purines and pyrimidines and generating more energy.

### Effects of drying methods on α-linolenic acid metabolism in Chrysanthemi Flos

In α-Linolenic acid metabolism, α-Linolenic acid can be liberated from the several complex fatty acids located mainly in the membranes of organelles such as chloroplasts. α-Linolenic acid modulates the transcription of genes implicated in diverse abiotic stress responses, including those to drought, salinity, and physical damage. α-Linolenic acid is involved in a number of pathways that are not only directly related to biological responses but also to a variety of abiotic stress conditions, suggesting that it plays a very important role as a signaling mediator [13]. α-Linolenic acid can be converted to 13(S)-hydroxylinolenic acid and 9(S)-hydroxylinolenic acid through a reaction catalyzed by LOX2S [14]. A recent study found that methyl jasmonate applied to post-harvest peaches mitigated their vulnerability to cold injury when stored at low temperatures [15]. Methyl jasmonate has been shown to stimulate α-linolenic acid metabolism, leading to enhanced membrane lipid unsaturation via a progressive reduction in unsaturated fatty acids (USFAs) coupled with a rise in saturated fatty acids (SFAs). α-Linolenic acid metabolism enhancement may contribute to the reduction in cold injury in peaches during cold storage. Intriguingly, we found that LOX2S was down-regulated in DCF vs. FCF. This may be due to the inhibition of LOX2S enzyme activity in Chrysanthemi Flos under high-temperature environments. Meanwhile, stearidonic acid was found to be down-regulated in both SCF and DCF, indicating that Chrysanthemi Flos reduces α-Linolenic acid consumption during drying. In plants under abiotic stress, lipids are broken down into free fatty acids [16]. As a free fatty acid, α-linolenic acid exhibits potent antioxidant properties and serves as a substrate for jasmonic acid production, which in turn functions as a signaling molecule, triggering subsequent stress mitigation pathways [17]. Chrysanthemi Flos resisted abiotic stress by maintaining a high concentration of α-Linolenic acid under the two drying methods, which was confirmed by the results of metabolite NO. 1457 (α-Linolenic acid) expression trend in FCF, SCF and DCF groups in Table S1.

### Effects of drying methods on amino acid metabolism in Chrysanthemi Flos

Amino acid metabolism is intricately linked with the dynamics of energy and carbohydrate fluxes, carbon and nitrogen allocation, and the biosynthesis of proteins and secondary metabolites [18]. According to our proteomics and metabolomics studies, glycine, serine and threonine metabolism and cyanoamino acid metabolism, namely, the amino acid metabolic pathways of Chrysanthemi Flos, were significantly affected when the flowers were processed using shade drying and oven drying.

Regarding glycine, serine, and threonine metabolism, threonine showed different degrees of down-regulation in both SCF vs. FCF and DCF vs. FCF. AGT3 was up-regulated in SCF vs. FCF, and GCSH was down-regulated in DCF vs. FCF. Threonine is an obligatory (amino acid) metabolite that is readily interconnected with methionine and isoleucine and reciprocally converted with glycine and serine [19, 20]. Threonine signals the plant's own receptors and converts them to specific functional outputs (for example, changes in plant growth, development, and metabolism, the expression of storage protein genes, abiotic stress tolerance, cell growth and division) when the plants are subjected to unfavorable conditions (e.g., abiotic stress tolerance) and other physicochemical factors [21, 22]. As shown in Fig. 5, threonine was lower in both DCF and SCF than in FCF; meanwhile, AGT3 was up-regulated, which may be due to the fact that the drying environment caused Chrysanthemi Flos to produce more threonine for resisting this stress on the one hand, and on the other hand, as one of the metabolic branches of the aspartic acid family pathway, threonine can be easily converted to glycine for subsequent amino acid metabolism, which provides nutrients for protein synthesis, secondary metabolism and other life activities [19, 23]. We found that GCSH, an aminomethyl-carrying intermediate in the glycine cleavage system (GCS) that carries hydrogen through the thiooctanoyl portion of the prosthesis, was down-regulated in SCF vs FCF [24]. The GCS comprises the T-protein, P-protein, L-protein, and H-protein, though the stability of their complex formation remains a subject of debate [25, 26]. Critical for photorespiration in C_3_ plants and for a recovery pathway in photosynthesis, the GCS's activity is notably low in senescing pea leaves but can surge by an order of magnitude upon light exposure [26]. When Chrysanthemi Flos is in a dry environment sheltered from light, the down-regulation of GCSH may be due to the inhibition of the glycine cleavage system reaction in SCF vs. FCF.

Cyanoamino acid metabolism is positively correlated with biotic stress tolerance [27]. L-isoleucine and L-valine are nutrients in the branched-chain amino acid (BCAA) group that are essential for humans and animals [28]. BCAAs play a key role as an osmotic regulator in plant stress tolerance [29]. In a study of *Arabidopsis thaliana’s* tolerance to drought stress, the branched-chain amino acid catabolic pathway was found to be required for its dehydration tolerance process [30]. In our study, isoleucine and valine showed various degrees of down-regulation in SCF vs. FCF and DCF vs. FCF, and it might be the case that these amino acids can be most efficiently used as alternative respiratory substrates during gradual senescence to death or carbohydrate starvation in Chrysanthemi Flos [31]. Meanwhile, the proteomics data indicated that β-glucosidase was down-regulated in DCF vs. FCF, potentially due to the heightened catabolic activity of branched-chain amino acids during high-temperature drying. The metabolomic data affirmed that more lotaustralin and linamarin were synthesized to combat this environment.

### Effects of drying methods on sugar metabolism in Chrysanthemi Flos

In plants, sucrose constitutes the principal photosynthetic output, acting as a crucial substrate for energy and a regulatory signal that modulates plant development. Additionally, sucrose plays a crucial role in responding to various abiotic stresses [32]. Exposure of Chrysanthemi Flos to desiccation stress under low or high thermal conditions alters the plant's carbon allocation and metabolism, leading to a depletion of energy reserves and a consequent decline in yield [33]. The accumulation of sugars in response to drought may influence the regulation of sugar and carbohydrate metabolism as well as their translocation within the plant [34, 35]. Our research indicates that the dehydration process markedly affects the dynamics of the starch and sucrose metabolism in Chrysanthemi Flos. Moreover, sucrose metabolism is crucial not only for plant development but also for abiotic stress responses [36, 37]. SUS was significantly up-regulated in SCF vs. FCF, while INV was markedly down-regulated in DCF vs. FCF. Furthermore, the differences between trehalose and inulin were also observed in SCF and DCF.

Starch and sucrose metabolism are pivotal in the growth and developmental processes of plants. The assimilation of sucrose into cellular metabolism is facilitated by two key enzymes: SUS and sucrose convertase [38]. Acting as a glycosyltransferase, SUS plays an integral role in the synthesis of starch and proteins, as well as in the generation of energy, predominantly within storage tissues [34, 35]. It catalyzes the reversible conversion of sucrose into fructose and nucleotide diphosphate glucose, either uridine diphosphate glucose (UDP-G) or adenosine diphosphate glucose (ADP-G). The metabolites produced through SUS-mediated sucrose breakdown are crucial for a host of metabolic functions, including energy generation, primary metabolite biosynthesis, and the formation of complex carbohydrates [39]. Research examining the effects of water deficit on the accumulation, translocation, and breakdown of carbohydrates in the foliage and roots of two soybean cultivars with comparable fertility levels revealed a notable elevation in SUS activity under conditions of water scarcity [32]. Moreover, it has been observed that SUS exhibits optimal activity at a temperature of 37 °C and maintains stability up to 50 °C [40]. The heightened activity of SUS in SCF relative to FCF may be ascribed to the enhanced enzymatic function in response to cooler, arid conditions.

In plant storage tissues, sucrose can be cleaved by both SUS and INV. SUS catalyzes the cleavage of sucrose into glucose and fructose in a reaction that is generally irreversible under physiological conditions [35]. In contrast, INV mediates a reversible reaction that converts sucrose and UDP into UDP-glucose and fructose. Sucrose requires more ATP to enter metabolism via INV than via SUS, providing more opportunities to coordinate this process with the carbon requirements of cellular metabolism [41]. Notably, abiotic stress suppresses the expression and activity of INV. Severe stress can cause plants to cease photosynthesis entirely, reducing the translocation of sucrose to the reservoir organs and leading to the repression of certain INV genes [35, 42]. The impact of drought stress on INV activity in soybean pods was confirmed by a study [43]. This finding is consistent with DCF where INV was down-regulated. Alginate, an intermediate product of starch and sucrose metabolism, plays a crucial role in reducing damage [44]. The marked up-regulation in alginate observed in DCF vs. FCF may be due to the fact that Chrysanthemi Flos reduces environmental damage to itself by increasing sugar metabolism under high-temperature conditions.

Sucrose must be broken down by the SUS enzyme to UDP-G and fructose, or by the INV enzyme to glucose and fructose. The need for these two sugar-degrading enzymes in plants is still not fully understood [39], as they may have different or complementary functions and energy requirements. SUS requires less energy for degradation than INV. It is common for them to operate in an apparently inefficient cycle, which could be significant for facilitating sucrose import and utilization or as a potential source of inefficiency [45]. Furthermore, both forms of sucrose catabolism present a promising energy source for energy production in Chrysanthemi Flos in response to various factors such as abiotic stress and metabolic regulation [46].

### Effects of drying methods on phenylpropanoid biosynthesis in Chrysanthemi Flos

Chrysanthemi Flos contains abundant flavonoids, phenylpropanes, and other phenolic compounds, of which flavonoids and phenylpropanes are the main active components, with biological functions such as anti-inflammatory, antimicrobial, antioxidant, blood-pressure-lowering, cholesterol-metabolism-accelerating, and immune-modulating activities [2]. Phenylpropanoid biosynthesis is a crucial secondary metabolic pathway in Chrysanthemi Flos, contributing significantly to its growth, development, and interactions with the environment [47]. The reactions initiating phenylpropanoid biosynthesis are PAL, C4H, and 4CL, catalyzing phenylalanine ammonia-lyase, cinnamate 4-hydroxylase, and 4-coumarate-CoA ligase. Generally, phenylalanine, which is the initial substrate of phenylpropanoid biosynthesis, undergoes conversion to cinnamic acid via PAL. Cinnamic acid is transformed into p-coumaric acid by C4H and subsequently activated by 4CL, yielding p-coumaroyl-CoA. This compound generates precursors for various downstream branches of the metabolic pathway [48]. Our discussion emphasizes the lignin and flavonoid pathways, which are the two primary branches of phenylpropanoid biosynthesis.

In our investigation, a comparative analysis between SCF and DCF against FCF revealed that within the phenylpropanoid biosynthetic pathway, the levels of sinapic acid and sinapyl alcohol were suppressed, while caffeoyl shikimic acid concentrations increased. Concurrently, the expression of enzymes PAL, 4CL, β-glucosidase, CAD, and POD inhibited, whereas F5H, HCT, and CCoAOMT enhanced significantly. Corroborating these findings, another study demonstrated that the spike organ of wheat under drought stress exhibited enhanced tolerance associated with the phenylpropanoid pathway, evidenced by elevated activities of PAL, C4H, and 4CL, alongside an increase in the antioxidant enzyme POD's activity [49]. In addition, the lignin synthesis pathway plays a key role under low temperature [50].

The complex regulatory mechanism of phenylpropanoid biosynthesis in Chrysanthemi Flos varies under different processing modes, as depicted in Fig. 5. PAL, the initial enzyme in the phenylpropanoid pathway, mediates the diversion of metabolites from the shikimic acid pathway to various phenylpropanoid branches by facilitating the synthesis of trans-coumaroyl-CoA from phenylalanine [51]. In contrast, 4CL facilitates the generation of p-coumaroyl-CoA in an ATP-dependent manner. Additionally, it promotes the attachment of multiple phenylpropanoid compounds to CoA, and various 4CL homologs display varying enzymatic preferences for distinct phenylpropanoid substrates [52]. Both light quality and intensity affect the expression of the genes responsible for phenylpropanoid biosynthesis in plants, with UV light exhibiting more significant inducible effects on PAL genes than white light [53]. PAL and 4CL also show clear co-expression characteristics [54]. In our study, PAL and 4CL were down-regulated in SCF vs. FCF and DCF vs. FCF, possibly attributed to light avoidance inhibiting the expression of PAL and 4CL in Chrysanthemi Flos. Additionally, CAD is integral to lignin biosynthesis, catalyzing the transformation of cinnamic aldehyde to its alcohol form, a process that contributes to lignin's accumulation and structural heterogeneity. Meanwhile, POD facilitates the last stage of lignin biosynthesis by promoting the polymerization of lignin’s phenylpropanoid precursors [55]. Both CAD and POD were down-regulated in SCF vs. FCF and DCF vs. FCF, which may potentially lead to the gradual senescence and eventual death of Chrysanthemi Flos. However, it is noteworthy that sinapyl alcohol showed an up-regulation in SCF vs. FCF, which contrasts with the trend of down-regulation observed in CAD expression in SCF. Although CAD is identified as the main enzyme catalyzing the biosynthesis of monolignol, SAD (sinapyl alcohol dehydrogenase) is actually involved in lignin biosynthesis [56, 57]. When CAD is down-regulated, its function may be compensated by an enzyme with similar activity. Preisner et al. utilized semi-quantitative PCR to analyze the gene expression in vitro cultures of CAD-reduced flax [57]. They found that CAD silencing resulted in a decrease in the activity of CAD genes in CAD27 and CAD33. Surprisingly, rational overexpression of the SAD gene compensated for the effects of CAD silencing on flax. The trend towards up-regulation of sinapyl alcohol, a CAD-regulated downstream metabolite, in SCF may be attributed to the compensatory mechanism of SAD. Meanwhile, the F5H enzyme, which is the third P450 enzyme involved in lignin monomer biosynthesis, facilitates nicotinamide adenine dinucleotide phosphorylation and the O_2_-dependent hydroxylation of a range of phenylpropanoid metabolites [58, 59]. The up-regulation of F5H was observed in both SCF and DCF, suggesting that this change may enhance the ability of Chrysanthemi Flos to resist the abiotic stresses that increase the synthesis and consumption of sinapic acid. Notably, the metabolic pathway from Coumaroyl-CoA to Sinapoyl-CoA showed an up-regulation of both metabolites and proteins. HCT, a bifunctional enzyme, facilitates the partial transfer of caffeoyl back to CoA, directing metabolic flow away from the general phenylpropanoid pathway towards lignin monomer biosynthesis [60, 61]. Additionally, CCoAOMT catalyzes methyl transfer reactions in lignin monomer biosynthesis [62]. Interestingly, HCT can cause a shift in metabolic flow towards flavonoid biosynthesis by activating lignin biosynthesis. This highlights HCT's key function as a gateway enzyme for lignin biosynthesis [63, 64]. We hypothesize that Chrysanthemi Flos will primarily exhibit resistance to low-temperature drying or high-temperature arid environments through this pathway during drying.

Flavonoid synthesis represents a significant segment of the phenylpropanoid pathway, yielding a vast array of polyphenolic compounds. Characterized by a C6–C3–C6 skeleton that includes two aromatic rings (A and B) and a central heterocyclic pyran ring (C), these metabolites are pivotal in plant defense, offering protection against various biotic and abiotic challenges including UV-B radiation, cold stress, and water scarcity [65]. There are two forms of CHI present in plants, and type I CHIs are found in Chrysanthemi Flos. They primarily convert chalcone to flavanones using 6′-hydroxychalcone as a substrate [66, 67]. Our investigation revealed that Chrysanthemi Flos’ flavonoid biosynthesis was active during processing, resulting in the up-regulation of CHI. The hydroxylation of flavonoids was found to improve their metabolic stability, membrane permeability, solubility, and antioxidant properties [68]. This could be because Chrysanthemi Flos promote the hydroxylation of flavonoids during drying to counteract their own negative effects [69]. In summary, phenylpropanoid biosynthesis plays a crucial role in the growth and development of plants as well as their interactions with the environment. Phenolic compound synthesis in plants escalates as a ubiquitous defensive response to stress, providing a shield against a spectrum of abiotic threats [70]. These compounds also play a vital role in a host of physiological functions, enhancing the plant's resilience and adaptive capacity to less-than-ideal environments [71–73]. The phenolic compounds in Chrysanthemi Flos contribute significantly to the plant’s resistance to the drying environment. However, as a natural consequence of harvesting, the inflorescences of Chrysanthemi Flos are detached from the chrysanthemum plant, leading to their eventual demise as discrete organs.

## Conclusions

In this study, we investigated the protein and metabolite changes in Chrysanthemi Flos under two different drying methods: shade-drying and oven-drying. Our findings suggest that the processing of Chrysanthemi Flos significantly impacts various metabolic pathways, including nucleotide metabolism, α-linolenic acid metabolism, amino acid metabolism, sugar metabolism, phenylpropanoid biosynthesis, and flavonoid biosynthesis. In the primary metabolic pathways, nucleoside metabolites (such as Guanine, Xanthine, and cytidine 5'-diphosphate), amino acid metabolites (such as serine, L-isoleucine, and stearidonic acid), and sugar metabolites (such as alginate and inulin) provide energy for the response of Chrysanthemi Flos to drought stress. 

Among the secondary metabolic pathways, phenylpropanes exhibited increased expression of F5H, HCT, CCoAOMT, and CHI enzymes in the flavonoid biosynthesis, suggesting that the drying process of Chrysanthemi Flos promotes the production of flavonoids with antioxidant effects, such as sinapic acid, caffeoyl shikimic acid, and naringenin chalcone. Furthermore, the shade-drying processing method is more conducive to retaining the active ingredients. However, despite the significant increase in energy metabolism and antioxidant capacity, the aging process in Chrysanthemi Flos was not prevented, ultimately leading to death. In summary, these findings offer a comprehensive understanding of the metabolite conversion and protein metabolism processes in SCF and DCF, providing valuable insights for optimizing the drying methods of Chrysanthemi Flos. Future investigations should prioritize examining the impact of Chrysanthemi Flos' endogenous enzymatic proteins on its chemical composition and regulatory mechanisms. This will provide a deeper understanding of the intricate metabolic regulatory processes that occur during Chrysanthemi Flos processing.

## Supplementary information

Additional file 1.

Additional file 2.

Additional file 3.

Additional file 4.

Additional file 5.

Additional file 6.

Additional file 7.

Additional file 8.

Additional file 9.

Additional file 10.

Additional file 11.

## Data Availability

The data in this study are available from the corresponding author upon reasonable request.
