# Supplementary material for: Metabolomics and proteomics analyses of Chrysanthemi Flos: a mechanism study of changes in proteins and metabolites by processing methods
Source: Chin Med. 2024 Nov 19;19:160. doi: 10.1186/s13020-024-01013-w (PMC11575428; doi:10.1186/s13020-024-01013-w)
Supplement: Supplementary file 1 — Additional file 1. [file 13020_2024_1013_MOESM1_ESM.docx]

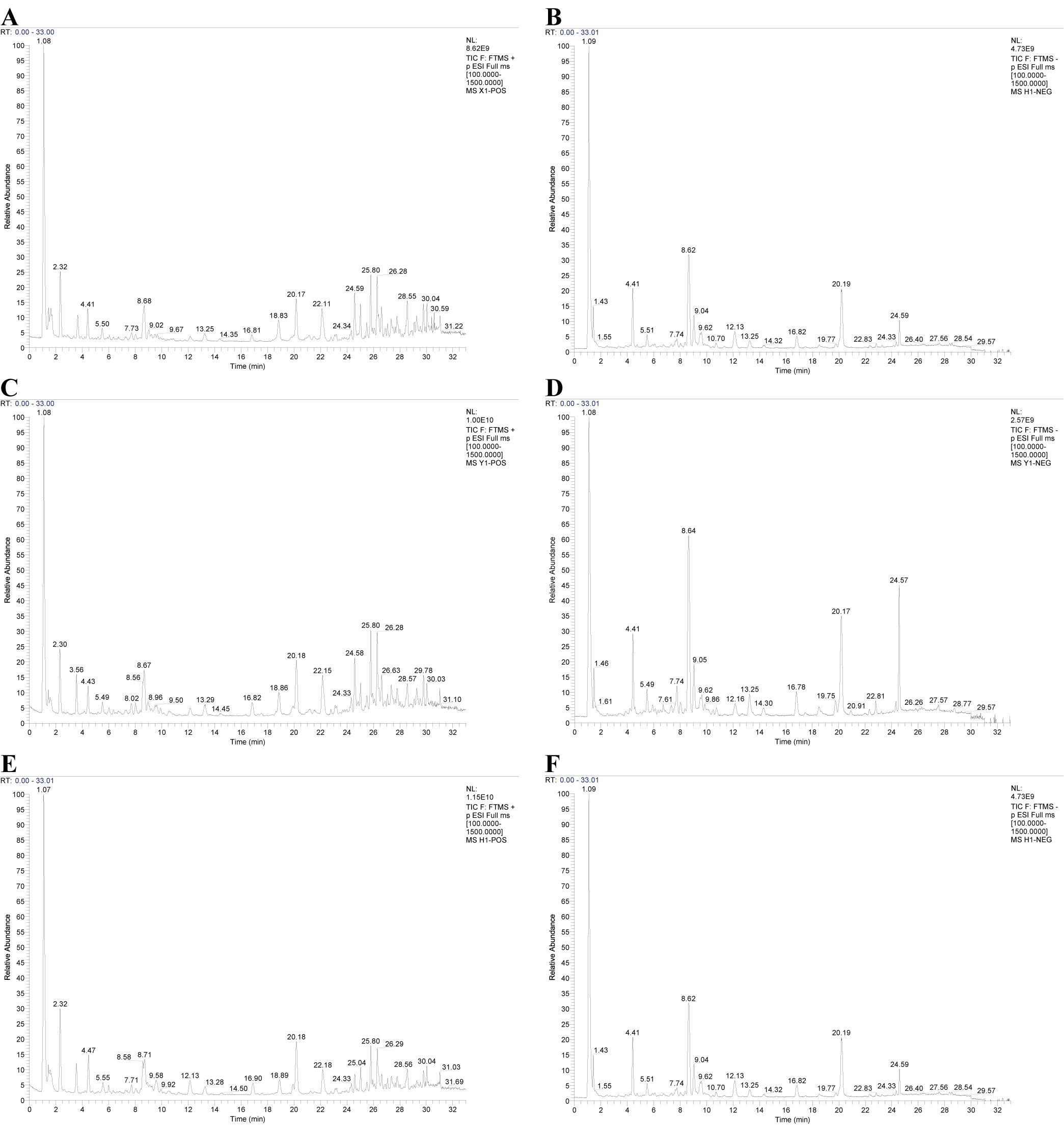


**Fig. S1.** The TIC diagram of Chrysanthemi Flos. A: Total ion chromatogram of FCF (+ESI mode); B: Total ion chromatogram of FCF (-ESI mode); C: Total ion chromatogram of SCF (+ESI mode); D: Total ion chromatogram of SCF (-ESI mode); E: Total ion chromatogram of DCF (+ESI mode); F: Total ion chromatogram of DCF (-ESI mode).
