# Supplementary material for: Metabolomics and proteomics analyses of Chrysanthemi Flos: a mechanism study of changes in proteins and metabolites by processing methods
Source: Chin Med. 2024 Nov 19;19:160. doi: 10.1186/s13020-024-01013-w (PMC11575428; doi:10.1186/s13020-024-01013-w)
Supplement: Supplementary file 2 — Additional file 2. [file 13020_2024_1013_MOESM2_ESM.docx]

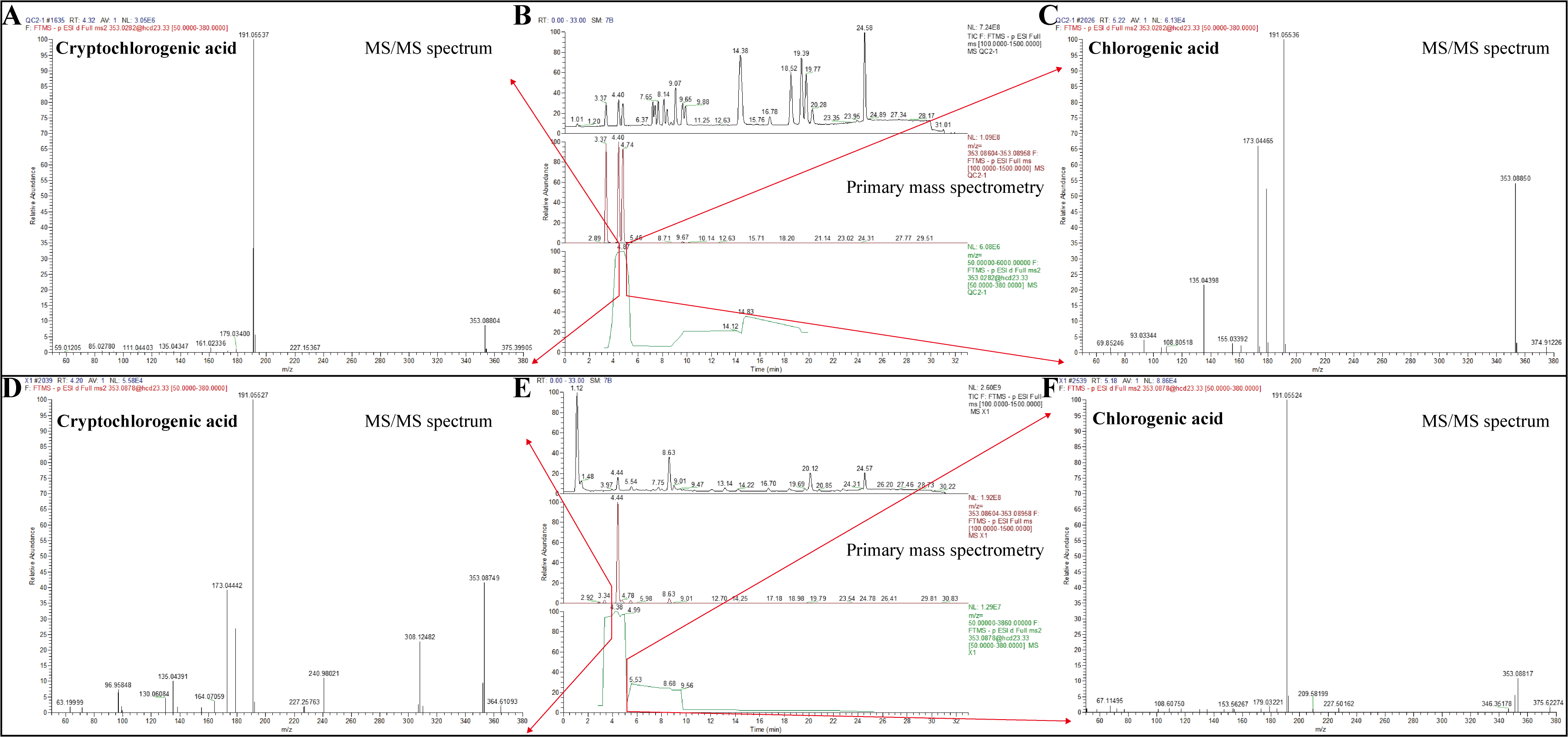


**Fig. S2.** Accuracy validation of metabolite identification. A: The MS/MS spectrum of the standard Cryptochlorogenic acid in QC sample. B: Detailed information of the primary mass spectrum of the standard Cryptochlorogenic acid and Chlorogenic acid from QC sample. C: The MS/MS spectrum of the standard Chlorogenic acid in QC sample. D: The MS/MS spectrum of Cryptochlorogenic acid in sample. E: Detailed information of the primary mass spectrum of the Cryptochlorogenic acid and Chlorogenic acid from sample. F: The MS/MS spectrum of Chlorogenic acid in sample.
