# Supplementary material for: Metabolomics and proteomics analyses of Chrysanthemi Flos: a mechanism study of changes in proteins and metabolites by processing methods
Source: Chin Med. 2024 Nov 19;19:160. doi: 10.1186/s13020-024-01013-w (PMC11575428; doi:10.1186/s13020-024-01013-w)
Supplement: Supplementary file 3 — Additional file 3. [file 13020_2024_1013_MOESM3_ESM.docx]

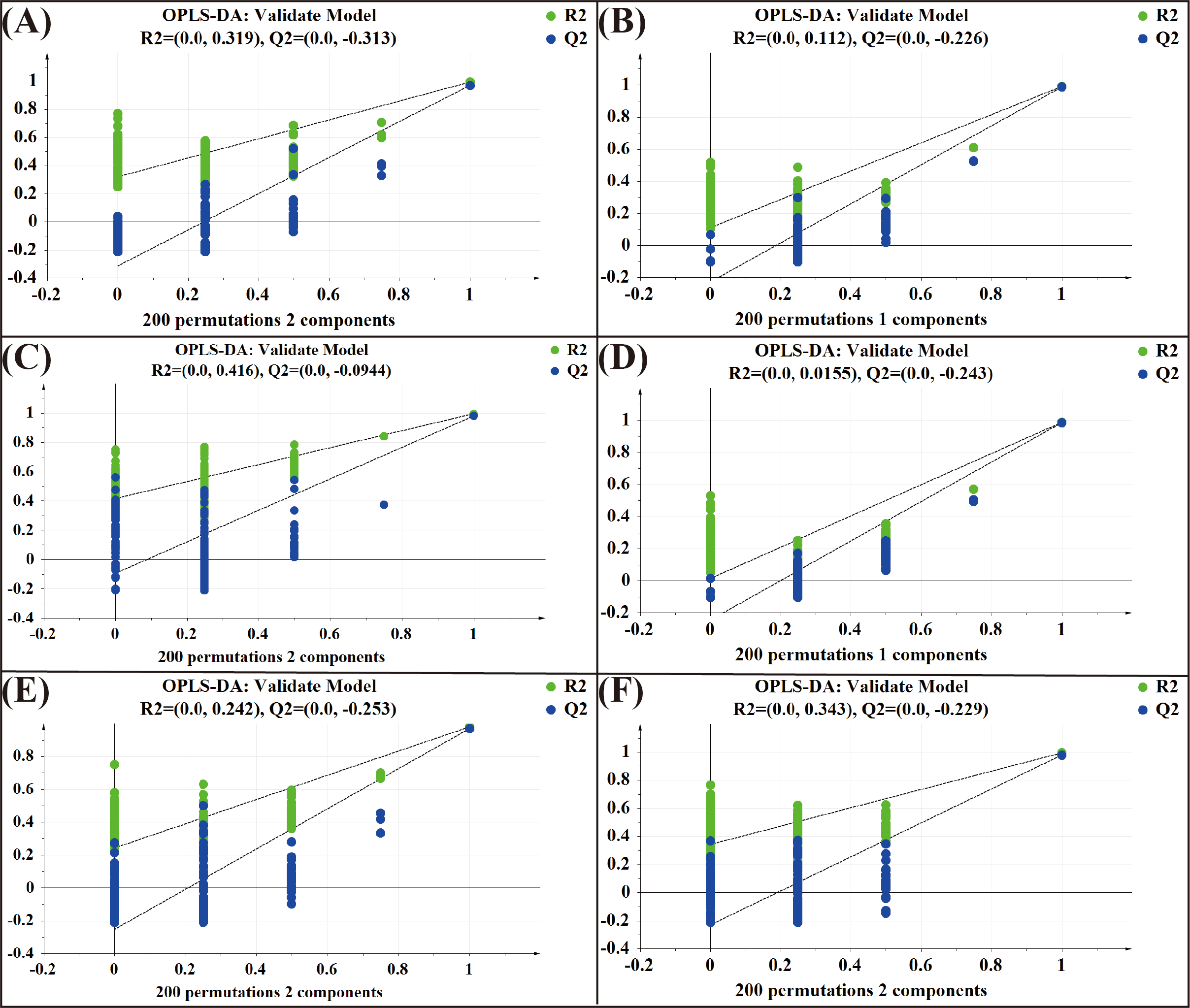


**Fig. S3.** 200-permutation test of OPLS-DA model. A–B: 200 permutation test of the FCF vs. SCF group in -ESI, +ESI, respectively. C–D: 200-permutation test of the FCF vs. DCF group in -ESI, +ESI, respectively. E–F: 200-permutation test of the SCF vs. DCF group in -ESI, +ESI, respectively.
