# Supplementary figures and images for: Metabolomics and proteomics analyses of Chrysanthemi Flos: a mechanism study of changes in proteins and metabolites by processing methods
Source: Chin Med. 2024 Nov 19;19:160. doi: 10.1186/s13020-024-01013-w (PMC11575428; doi:10.1186/s13020-024-01013-w)

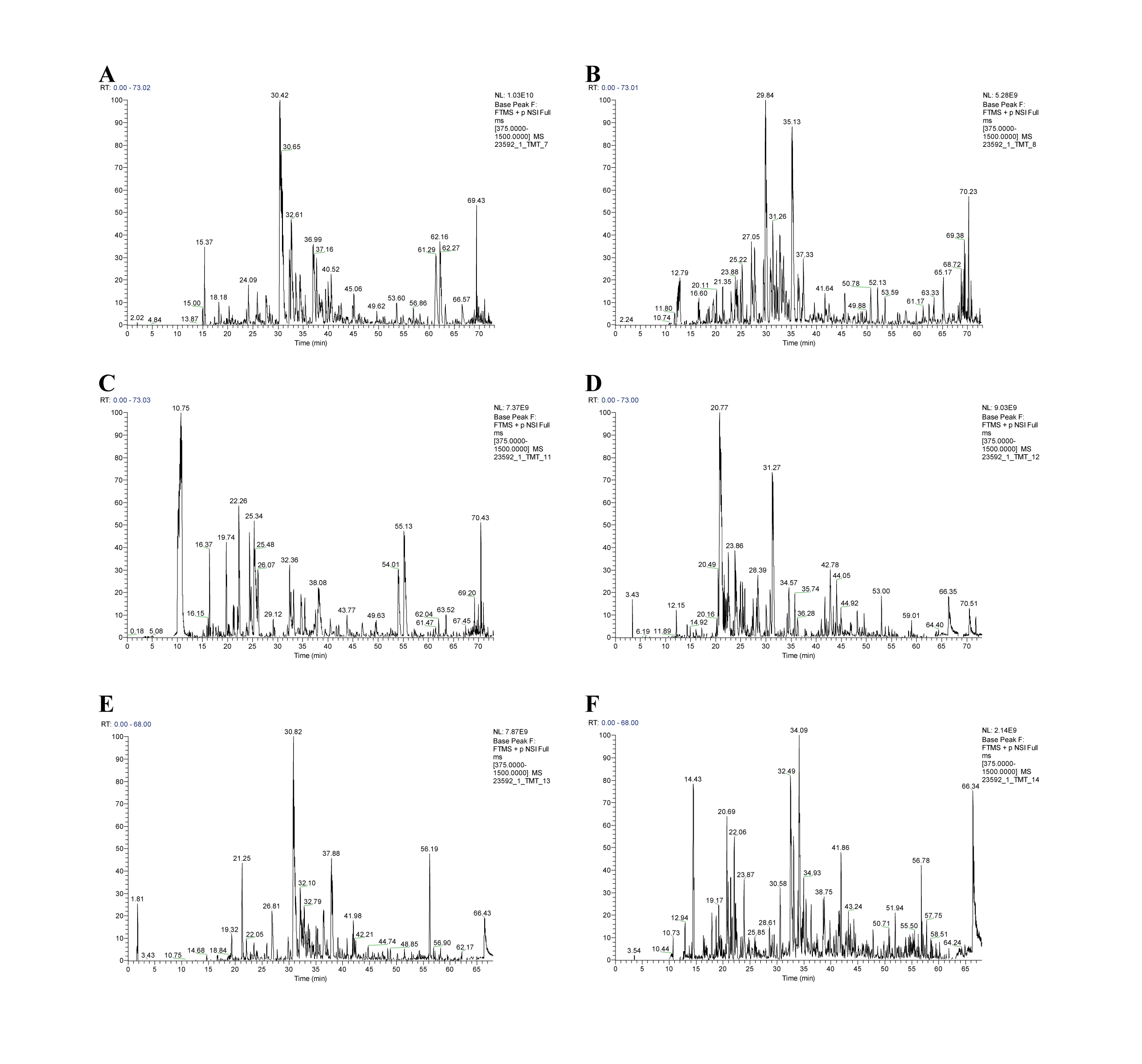
**Fig. S4** Chrysanthemi Flos samples protein mass spectrum.

Supplement: Supplementary file 4 — Additional file 4. [file 13020_2024_1013_MOESM4_ESM.docx]

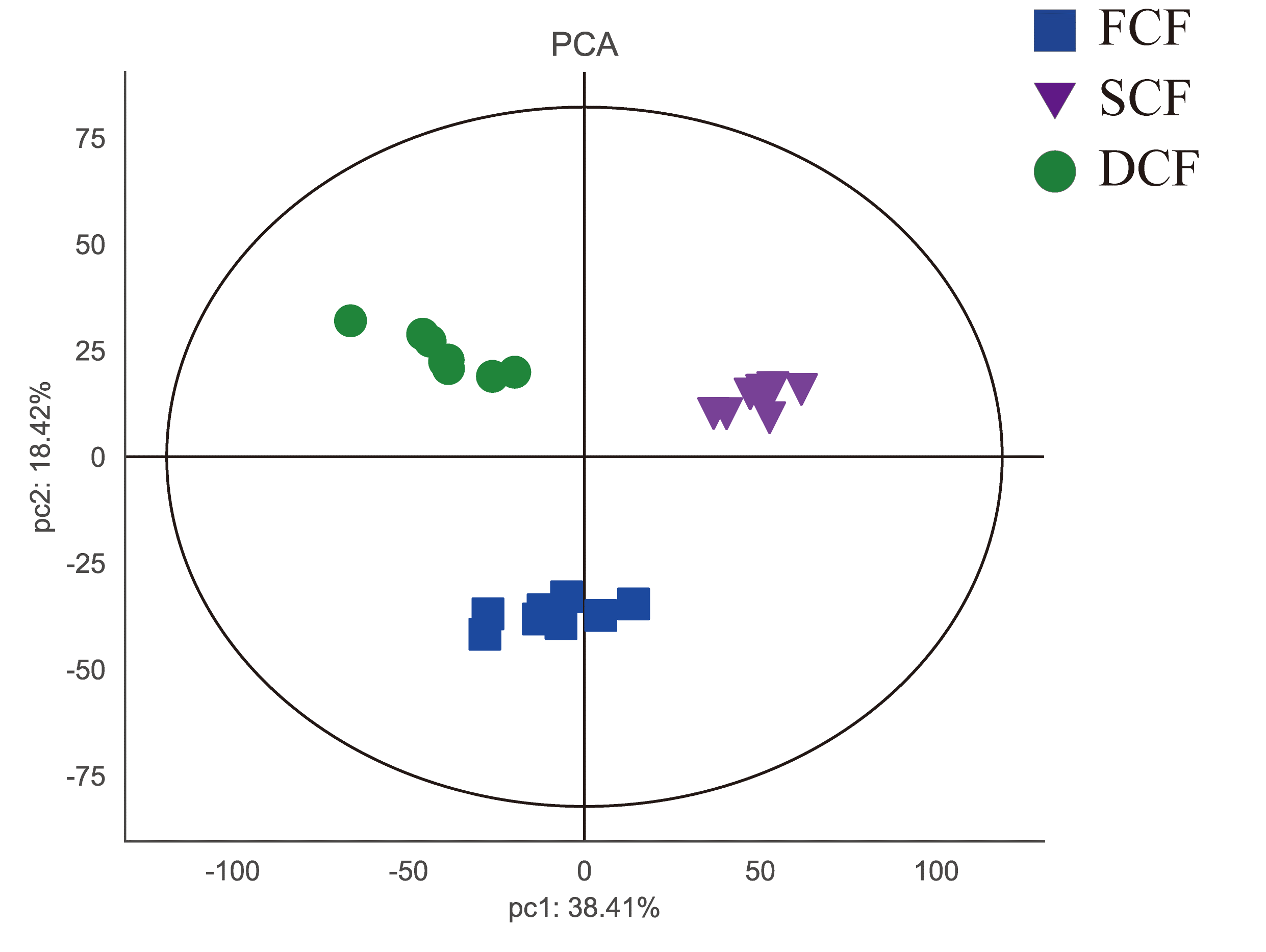
**Fig. S5** PCA results of proteomics.

Supplement: Supplementary file 5 — Additional file 5. [file 13020_2024_1013_MOESM5_ESM.docx]
